# Saudi Honey: A Promising Therapeutic Agent for Treating Wound Infections

**DOI:** 10.7759/cureus.18882

**Published:** 2021-10-19

**Authors:** Abdulrahman S Bazaid, Abdu Aldarhami, Hattan Gattan, Bakheet Aljuhani

**Affiliations:** 1 Clinical Laboratory Sciences, College of Applied Medical Sciences, University of Hail, Hail, SAU; 2 Medical Microbiology, Qunfudah Faculty of Medicine, Umm Al-Qura University, Al-Qunfudah, SAU; 3 Medical Laboratory Sciences, King Abdulaziz University, Jeddah, SAU; 4 Medical Laboratory Sciences, University of Hail, Hail, SAU

**Keywords:** alternative therapies, saudi honey, manuka honey, antimicrobial resistance, wounds infections

## Abstract

Treatment of wounds, especially chronic ones, is a major challenge in healthcare, with serious clinical and economic burdens. Multiple treatment approaches, including the usage of silver and iodine, have dramatically improved wound healing and reduced the incidence of infection. However, once infected by drug-resistant bacteria, treatment of wounds becomes a serious complication, with limited availability of effective antibiotic drugs, leading to high morbidity and mortality. Therefore, alternative therapeutic agents are required to address this gap in wound management. The introduction of manuka honey as a therapeutic agent against infected wounds was the result of extensive research about its activity against both planktonic and biofilm bacterial growth. Likewise, several types of Saudi honey (e.g., Sidr and Talh) showed promising in vitro and in vivo antimicrobial activity against wound pathogens. This short review summarizes literature that investigated the activity of common types of Saudi honey in relation to wound infections and explores their clinical utility.

## Introduction and background

Wounds can be defined as breakage or injury of the (superficial or deep) layers of the skin, leading to loss of its integrity and function [1]. Generally, wounds are the outcome of physical, mechanical, or thermal injuries [2]. Clinically, wounds are classified as either acute or chronic, depending on the duration and sequence of healing [1]. Acute wounds heal in up to four weeks and the healing process is defined as follows: inflammation, matrix precipitation, wound contraction, and epithelialization [3]. In contrast, chronic wounds do not follow this healing process and have an extended healing time [1]. Chronic wounds, which are commonly associated with high mortality rates, are triggered by underlying pathologies, such as diabetes, which may cause persistent inflammation [4].

Wound infections

Bacterial colonization of wounds can sometimes be observed without causing an infection [5]. This is because the initiation of bacterial infections within wounds depends on the bacterial load, adaption to the wound conditions, and their respective virulence factors [5]. Additionally, while the diversity of skin microbiota at the site of the wound is altered, whether or not, this contributes to delayed healing is still inconclusive [6]. Wound infections can be caused by Gram-positive (e.g., Staphylococcus aureus and Streptococcus pyogenes) and Gram-negative (e.g., Pseudomonas aeruginosa, Escherichia coli, and Serratia marcescens) bacteria, with varying severity. Infection with S. pyogenes is always considered of high concern due to its serious damage to the skin, known as necrotizing fasciitis [7], while both S. aureus and S. pyogenes have certain virulence factors (e.g., hemolysins) that are associated with impaired wound healing [8,9]. Wound pathogens, including P. aeruginosa and S. aureus, are well-known for their ability to form biofilms through the use of surface proteins (e.g., protein F1/SfbI), which adhere to the host cells and enable bacterial aggregation [10]. Biofilms exhibit considerable resistance to antibiotics, which may impair wound healing and deteriorate the clinical outcome [10].

Treating chronic wounds is a major challenge in healthcare globally [1]. There is therefore a need for an urgent and effective therapeutic plan to facilitate wound management and limit potential complications [1]. Wound bed preparation (WBP), a recommended protocol based on clinical practice guidelines, is applied to remove non-viable tissue from a wound, manage exudate, and prevent the establishment of infections [11]. To achieve successful WBP, chronic wounds need to be cleaned with high-pressure water to debride them, as there is no evidence-based recommendation that the use of chemical solutions, such as decylenamidopropyl betaine, leads to better outcomes [11]. Moreover, maintaining a moist wound environment with hydrogels is important to accelerate epithelialization and prevent any further damage by blisters [12]. Systemic antibiotics should only be prescribed in the case of infected wounds, although prophylactic antibiotics are offered to all patients with surgical wounds [13]. Several studies have evaluated the use of topical antimicrobial dressings, such as silver and iodine, for the management of chronic wounds, which revealed a significant reduction in S. aureus bacterial load in infected wounds [14,15]. In addition, ionic silver in wound hydrogels has shown promising effects against both planktonic and biofilm bacterial growth [16]. However, resistance to silver was observed in certain wound-infecting bacteria, such as E. coli and Acinetobacter baumannii [17]. Manuka honey, as a natural alternative, was successful in preventing biofilm formation by P. aeruginosa and S. aureus and limited the possibility of developing resistance, making its potential therapeutic use in treating wound infections very promising [18-20].

Antimicrobial resistance

Since the discovery of penicillin by Alexander Fleming in 1928, the ongoing use and misuse of antibiotics have led to the emergence of antimicrobial resistance (AMR), where antimicrobial compounds have become less effective against resistant bacteria in the clinical setting [21]. The emergence of multi-drug resistant (MDR) organisms, those resistant to two or more antibiotics classes, has complicated the management of infected wounds [21], and therefore, alternative approaches, including the use of natural products, have been suggested as potential replacement or adjunct treatment with conventional antibiotics [22]. Searching for suitable and effective alternative therapeutic agents to treat infections associated with human wounds, especially those caused by MDR pathogens, has become a world health priority [19].

## Review

Honey as a wound remedy

Honey, as a natural product, has been used by different cultures as a wound remedy over many centuries [19,23], owing to its well-known antimicrobial activity [23]. The antimicrobial activity of honey is attributed to the effect of phenolics, osmatic pressure, and hydrogen peroxide (multiple modes of action), making bacterial resistance less likely to occur [23]. The potency and mode of action of different types of honey differ according to the geographical location, the type of plant used by the bees, and the concentration of the honey [24]. Certain physicochemical components of honey can trigger monocytes to release cytokines, such as interleukin (IL)-1 and IL-6, as well as tumor necrosis factor-alpha (TNF-α), which modulate the immune response to infections (immunomodulation effects) [25]. 

Recently, a type of honey from New Zealand, Manuka honey, was optimized for use as medical-grade honey for wound dressing [19]. This was the result of observed antimicrobial properties of methylglyoxal, which can promote wound healing and exhibit potent activity against various planktonic and biofilm-forming Gram-positive and Gram-negative pathogenic bacteria, including wound pathogens [19]. Interestingly and importantly, increased in vitro sensitivity was observed in bacterial strains treated with Manuka honey to clinically used antibiotics, compared with untreated bacteria [20].

A recent in vivo study has shown an interesting positive impact upon the healing process of chronic wounds when treated with medical-grade honey (MGH) while the absence of any antibiotic drugs in almost all patients (eight of nine cases) [26]. This is because wound odor, pain, exudation, and signs of wound infections were completely inattentive following the application of the MGH. In addition, the utilize of the MGH has endured an auto-debridement leading to a cleansed wound bed, and well-established granulation tissue and epithelialization. These findings surely supported the potential use of an optimized MGH to treat wound infections and facilitate its healing process.

Saudi honey

Saudi honey, e.g., Sidr (Ziziphus spina-christi) honey, showed promising in vitro results against pathogenic drug-resistant bacteria, such as *S. aureus*, *S. mutans*, *K. pneumonia,* and *E. coli* [27]. The most potent dilution of Sidr honey was determined as 20% (w/v) against *S. mutans* followed *E. coli*, *S. aureus*, and *K. pneumoniae* based on the optical density of tested bacteria before and after exposure to the honey as follows: 1.809 to 0.359, 1.528 to 0.411, 1.721 to 0.497 and 1.746 to 0.511, respectively [27]. Multiple in vitro studies on the antimicrobial activity of Saudi honey presented various activity profiles and potency levels against tested Gram-positive and Gram-negative pathogenic bacteria [28], according to the source of nectar, the season, and the geographical region [29]. In essence, Sidr and *Nigella sativa* types of honey (50% w/v) showed an in vitro bacteriostatic effect against wound isolates of imipenem-resistant and sensitive strains of *P. aeruginosa*, while the same concentration of Manuka honey was shown to have bactericidal activity against the same bacteria [30]. The activity of Sidr honey is variable across different harvest regions, and low concentrations (20% w/v) tend to be more active against Gram-positive than Gram-negative bacteria, while higher concentrations show a broader spectrum of activity [27]. Although no study has report bacterial resistance to honey, Gram-positive isolates of *Bacillus cereus* and *S. aureus* exhibited increased in vitro sensitivity to Sidr honey (33% w/v), evidenced by the large zone of inhibition (36 mm and 34 mm, respectively) compared to *E.coli* and *Salmonella enteritidis* (27 mm and 23 mm, respectively) [31]. This is in line with previous reports where the inhibition of Gram-positive bacteria (*Enterococcus faecalis* and *S. aureus*) at lower the concentration of Manuka honey was observed while its absent/limited effect on Gram-negative bacteria (*P. aeruginosa* and *E. coli*) [32].

Sidr honey from the Najran region has high in vitro antimicrobial activity (40% w/v) against *E. coli *(zone of inhibition 30 mm) compared to *P. aeruginosa* (zone of inhibition 18mm) and *A. baumannii* (zone of inhibition 19 mm) (Table 1) [33]. By contrast, Sidr honey from the Al-Hasa region showed no in vitro activity (50% w/v) against a wide range of Gram-positive (e.g.,* S. aureus*,* S. epidermidis*,* S. pyogenes*, and *B. subtilis*) and Gram-negative (e.g., *E. coli*,* P. aeruginosa*,* P. mirabilis*, and *S. enterica* Typhimurium) bacteria [34]. Therefore, a different or even a single variety of honey can have variable activity profiles while the observed potency of active products can be relatively similar to commonly used broad-spectrum antibiotics [31]. 

**Table 1:** Saudi Sidr honey from different regions and brands with various active concentrations against tested pathogenic bacteria. MRSA: methicillin-resistant *Staphylococcus aureus*; MSSA: methicillin-sensitive *Staphylococcus aureus*

Bacteria	Honey brand or region	Active concentration	Reference
Staphylococcus aureus	Alnahal Aljwal	21%	Hegazi et al., 2017 [27]
Streptococcus mutans			
Klebsiella pneumoniae			
Escherichia coli			
Pseudomonas aeruginosa			
Staphylococcus aureus	El-Yahia	20%	Hegazi, 2011 [28]
Streptococcus pyogenes			
Corynebacterium pseudotuberculosis			
Klebsiella pneumonia			
Pseudomonas aeruginosa			
Escherichia coli			
Pseudomonas aeruginosa	Valley honey	50%	Al-Nahari et al., 2015 [30]
Staphylococcus aureus	Middle and Northern regions	33%	Owayss et al., 2020 [31]
Bacillus cereus			
Escherichia coli			
Salmonella enteritidis			
Salmonella enteritidis	Taif	16%	Halawani et al., 2011 [35]
Escherichia coli	Asir	20%	Ghramh et al., 2019 [36]
Proteus mirabilis			
Staphylococcus aureus			
Staphylococcus epidermidis			
MSSA	Al-Baha	50%	Hussain et al., 2019 [37]
MRSA			
Escherichia coli	Asir	20%	Ghramh et al., 2020 [38]
Pseudomonas aeruginosa			

Talh (*Thymus vulgaris*) honey is another type of Saudi honey, with specific medicinal properties, including promising in vitro activity against pathogenic bacteria [27]. Similar to Sidr honey, Talh honey (33% w/v) is more potent against Gram-positive (*S. aureus* and *B. cereus*) than Gram-negative (*E. coli* and *Salmonella enteritidis*) strains (Table 2) [31]. A randomized controlled trial investigated the antimicrobial activity of Saudi Talh honey as a dressing agent against 20 diabetic foot wounds [39]. The use of Talh honey led to a significant reduction (100 fold) in bacterial count (9 x 10^2^ CFU) following day 17 of application, compared to bacterial load (9 x 10^4^ CFU) recorded after using dressing made of povidone-iodine and saline [39]. Moreover, inflammatory cytokines, mainly IFN-γ, IL-1β, and IL-6, were significantly suppressed (by 12%, 50%, and 55%, respectively), compared with the initial level of response prior to the use of honey [39]. These findings are a clear indication of the in vivo antimicrobial activity of Saudi Talh honey. This activity is often attributed to phenol and hydrogen peroxide, which have been found in high concentrations in these varieties of honey [35].

**Table 2:** Talh Saudi honey from different regions and brands with various active concentrations against tested pathogenic bacteria. MRSA: methicillin-resistant *Staphylococcus aureus*; MSSA: methicillin-sensitive *Staphylococcus aureus*

Bacteria	Honey brand or region	Active concentration	Reference
Staphylococcus aureus	Alnahal Aljwal	21%	Hegazi et al., 2017 [27]
Streptococcus mutans			
Klebsiella pneumoniae			
Escherichia coli			
Pseudomonas aeruginosa			
Staphylococcus aureus	Middle and Northern regions	33%	Owayss et al., 2020 [31]
Escherichia coli			
Salmonella enteritidis			
MSSA	Al-Baha	50%	Hussain et al., 2019 [37]
MRSA			

## Conclusions

Saudi honey has promising activity against a wide range of pathogenic bacteria, with potential use to treat wound infections. Although the activity and level of potency of the tested types of Saudi honey were variable, the most popular varieties of Saudi honey (Sidr and Talh) showed promising broad-spectrum bacteriostatic activity, which seemed to be more potent against Gram-positive bacteria. There remains a need for focused studies to evaluate the potential prophylactic use of Saudi honey against antibiotic-resistant and biofilm-forming bacteria in wounds. Further, systems biology studies to elucidate the impact of Saudi honey on planktonic and biofilm growth of various wound bacteria at the cellular and genomic levels are required. Such data may guide the selection of optimized Saudi honey with higher activity against pathogenic bacteria, with a role as medical-grade honey for treating wound infections.
